# Views from senior Australian cancer researchers on evaluating the impact of their research: results from a brief survey

**DOI:** 10.1186/s12961-015-0073-0

**Published:** 2016-01-12

**Authors:** L. G. Gordon, N. Bartley

**Affiliations:** 1 Menzies Health Institute Queensland, Griffith University, Centre for Applied Health Economics, University Drive, Meadowbrook, Logan, QLD 4131 Australia; 2 Cancer Council NSW, Research Strategy Unit, Woolloomooloo, Sydney, NSW 2001 Australia

**Keywords:** Cancer research, Research impact methods, Research policy

## Abstract

**Background:**

The interest and activity in measuring and reporting the impact of publicly funded health and medical research has grown rapidly in recent years. Research evaluation typically relies on researchers for much of the information for an impact assessment. However, the acceptability and feasibility of this activity among health researchers is unknown. The aim of this study was to understand the role and opinions of cancer researchers in the growing area of impact evaluation activity, to inform the logistics of a sustainable program of impact evaluation.

**Methods:**

A brief anonymous online survey was administered to 95 current and past grant recipients funded through the external grants program at Cancer Council New South Wales. Eleven survey statements were constructed with Likert responses and supplemented with two open-ended questions. The statements covered the conceptual, attitudinal and practical aspects of impact evaluation. The survey targeted researchers from the full spectrum of cancer control research classifications. Descriptive analyses obtained response frequencies and percentages.

**Results:**

Forty-five cancer researchers completed the survey (response rate 47%) and 77% were Associate Professors or Professors. Responses were polarised for questions relating to engaging with research end-users, perceived time-pressure to collate data, and pressure to produce research outputs. Some researchers emphasised that quality was an important goal over quantity and warned that collecting impact data created incentives and disincentives for researchers.

**Conclusion:**

There was mixed support and acceptance among senior cancer researchers in Australia on their perceived role and engagement with research impact activities. Sole reliance on researchers for collating and reporting impact data may be problematic. Requesting information from researchers could be minimised and confined to final reports and possible verification of externally-led evaluations.

## Background

In recent years there has been significant interest in measuring and reporting the impact of publicly funded health and medical research. This has occurred as funding agencies face increased pressure to demonstrate accountability and for informing future grant decisions [1]. Funding agencies also use impact assessments for advocacy purposes, clearly articulating research to the general population, and for internal analysis, to identify research strategies that are most likely to produce future benefit.

Capturing the outputs for individual research projects or a cohort of projects typically relies on principal researchers for much of the information. An impact assessment will usually include information not only for publications and academic research outputs but on less visible outputs such as building workforce capacity, supervision of PhD and masters students, patent applications, leveraged funds, evidence of health policy changes, wider clinical practice changes and so forth. The participation by the researcher is crucial and the researcher is essentially the central portal for this information [1]. Data on publications and research outputs are routinely collected through annual reporting processes which researchers are familiar and have high compliance with (sometimes mandated by the funder) [2]. Downstream impact data is less routinely collected and researchers are not as used to reporting this data. This data can be collected by surveys targeted at groups of researchers within an organisation over a set time period [3].

Although the principal researcher (grant recipient) offers a prime source of data on research outputs, difficulties in implementation of an impact assessment may mean they are not the obvious starting point for collection of impact data. Primarily the assessment is an administrative function and the burden to researchers for yet more non-research activity within their routine jobs is mounting [4]. The opportunity cost of spending time on this activity is the foregone activities of teaching, laboratory work or other core research priorities. Studies and reports on health and medical research impact assessment show that questionnaire methods asking researchers to provide output information (i.e. bibliometrics, academic outputs and policy impacts) have poor response rates [2, 3, 5]. Researchers may also provide incomplete data or, if tied to funding, they may be inclined to exaggerate their claims on impact and introduce a biased viewpoint. Conversely, other studies have shown from triangulation of data sources, that surveys of the lead investigators underestimate their impacts [3]. Researchers do not always communicate their outputs clearly and they may also be unaware of downstream events.

Ideally, organisations interested in research impact evaluation need to have engaged researchers and community members. In a recent Australian report, one of the conclusions was that “*there is a need, and an opportunity, to create and embed a culture of and expectation for impact within Australian universities and wider society*” [6]. However, beyond the small number of outspoken researchers who have submitted their opinions in various forums [4, 7, 8], there is a lack of understanding on what the broader research community attitudes and values towards research impact are. We undertook a brief survey aimed at principal cancer researchers across Australia who were funded through the external grants program at the Cancer Council New South Wales (CCNSW) in order to understand their role and sentiments in this growing area of impact work.

Understanding the views of cancer researchers is important as cancer is a leading cause of morbidity and mortality worldwide, and attracts significant research investment of research teams around the globe. In Australia alone, more than $1 billion was provided between 2006 and 2011 by all major cancer research funders to support cancer research and programs [9]. CCNSW is the largest non-government funder of cancer research in the state of New South Wales as well as Australia more broadly. Since 2006, CCNSW has committed close to $100 million in new funding through the external research program to researchers across Australia. Understanding the views of cancer researchers on evaluating the impact of their research allows us to better inform the development and implementation of the evaluation and monitoring framework used to measure research impact of CCNSW research funding.

## Methods

### Survey sample

With the assistance of the CCNSW Research Strategy Unit, the survey targeted current and past grant recipients identified by CCNSW records (n = 95). These included the principal researchers who had been or were involved in projects covering a range of competitive grant schemes administered by the CCNSW and strategic research partnership, program, project and innovator grants. It also covered the full spectrum of cancer control research as categorised using the Common Scientific Outcomes from biology through to scientific model systems.

### Survey content

The online survey was developed using the Qualtrics platform. The survey questions were developed by the authors and tested among colleagues with final approval from the CCNSW Research Strategy Unit. The content and wording of the survey questions were intended to be somewhat provocative so that respondents would avoid neutral or indifferent responses (Box 1). The survey covered both the conceptual (Questions 1–4), practical (Questions 5–7) and attitudinal (Questions 8–10) issues of research impact evaluation. The statements were developed by the authors and derived through reading of published papers and commentaries [2–5, 7, 8, 10] and reports [1, 6, 11, 12] on this topic. A short survey with closed and open-ended questions was considered the most appropriate method to obtain both the quantitative data regarding researchers’ attitudes and views, as well as qualitative data allowing further exploration of issues, taking into consideration the issue of time-poor senior researchers.

**Table 1:** Number of respondents by their career level and type of cancer research by Common Scientific Outcomes

	Post-doc <5 years	Post-doc 5+ years	Associate Professor	Professor	Total
Biology	0 (0%)	5 (63%)	6 (43%)	5 (26%)	16 (37%)
Aetiology	1 (50%)	0 (0%)	0 (0%)	1 (5%)	2 (5%)
Prevention	0 (0%)	0 (0%)	2 (14%)	2 (11%)	4 (9%)
Treatment	1 (50%)	2 (25%)	3 (21%)	8 (42%)	14 (33%)
Cancer control, survivorship, outcomes	0 (0%)	0 (0%)	2 (14%)	3 (16%)	5 (12%)
Scientific model systems	0 (0%)	1 (13%)	1 (7%)	0 (0%)	2 (5%)
Total	2 (5%)	8 (19%)	14 (33%)	19 (44%)	43^a^ (100%)

^a^Two respondents did not answer the questions of researcher level and type of research.

**Box 1:** **Survey questions**

Q1. I am conscious that, because I am paid with public/charitable funds, I need to deliver meaningful outcomes from my research.	
Q2. I should be accountable for the specified outputs of my research project/program but not for engaging with stakeholders or the end-users of the research.	
Q3. Research impact should only be based on measurable academic outputs, e.g. reports, publications, h index, citations, conference presentations, etc. (subject to their limitations).	
Q4. Measures of research impact should include the broader downstream influences on patients, improved health and well-being, healthcare delivery and improving society (subject to their limitations).	
Q5. For your type of research, what is/are the most appropriate measure(s) of research impact? (open-ended)	
Q6. I would like to have input into what is perceived as the impact of my research project or program and I accept that it is a necessary part of my role as a researcher.	
Q7. I spend far too much of my time collating evidence on the impact of my research at the expense of core research or teaching.	
Q8. I accept that it is part of my job as a grant reviewer to judge the potential significance of potential impact of a research proposal to assist the decision making process.	
Q9. I feel a lot of pressure to produce research outputs to the point that it is hampering my scientific freedom.	
Q10. Evaluating the outcomes of my research undermines my authority and autonomy at undertaking good science.	
Q11. The type of health or medical research that best describes my work is: biology, aetiology, prevention, treatment, cancer control/survivorship/outcomes, scientific model systems.	
Q12. I am at the following level of my career: PhD student, post-doctoral researcher for less than 5 years, post-doctoral researcher for more than 5 years, associate professor, professor.	
Q13. Additional comments (open-ended).	
*Responses to all questions except open-ended Questions 5 and 13 used a Likert scale; ‘strongly agree’, ‘agree’, ‘neither agree or disagree’, ‘disagree’, and ‘strongly disagree’*	

In total, 11 statements were provided with responses using a Likert scale; ‘strongly agree’, ‘agree’, ‘neither agree or disagree’, ‘disagree’, and ‘strongly disagree’ (Box 1). Question 5 was open-ended and specifically asked what impact measures should be used as most relevant for the cancer researcher’s work. The final question was open-ended and intended to elicit other respondent concerns or comments that were not already covered. The survey was planned to take no more than 5 minutes but with the addition of the final open question, may have taken longer depending on respondent input. There was no word limit for the open-ended questions. Two additional questions, the type of cancer research (i.e. biology, aetiology, treatment, prevention, cancer control/survivorship/outcomes, scientific model systems) and researcher career level (i.e. professor, associate professor, post-doctoral researcher for at least five years or less than five years), were asked in order to understand how these attributes influenced the responses.

### Recruitment and data collection

The principal investigators were emailed an invitation to complete the survey via a hyperlink. To maximise response rates, the survey responses were anonymous and no identifying information was collected that could be traced back to the individual cancer researcher. Ethics approval for the survey was received by the Griffith University Human Research Ethics Committee.

The survey was open and accessible to respondents from 11th June to 4th July 2014 (3½ weeks). Two reminder emails were sent during this time to prompt further completions. After a total of three emails, no further responses were received and the survey was closed. Descriptive analyses were performed to obtain the counts and percentages of responses. Cross-tabulations and χ^2^ tests were planned for subgroup analyses by researcher career level and type of research.

## Results

**Fig. 1:**
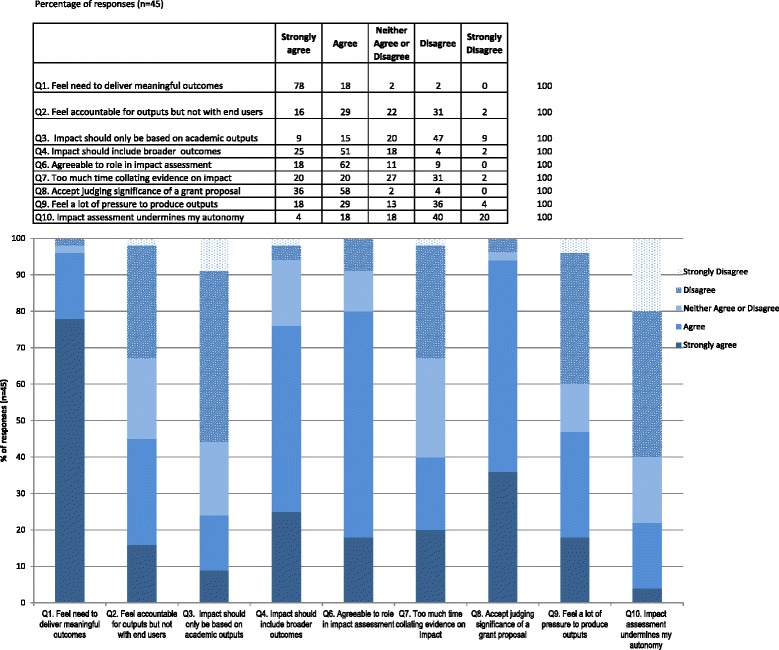
**Results of survey.**

In total, 45 out of 95 cancer researchers completed the survey (response rate 47%). Most respondents were professors 19 (44%) or associate professors 14 (33%), and the most common types of research were biology 16 (37%) and treatment 14 (33%) (Table 1). The results of the closed questions are presented in Figure 1.

Overall, there was clear consensus among respondents for some questions but substantial variability for others. Respondents had a keen sense of social responsibility to deliver meaningful outcomes from their research activities (Question 1, 35/45 or 78% strongly agreed). There were different views about their role in being accountable to end-users of the research (Fig. 1, Question 2). Although the question did not define end-users or stakeholders specifically, many disagreed this was a researcher’s role. This raises the issue that some researchers do not expect or feel the need to communicate their scientific work with the broader community. Whether this task is seen as difficult, too time consuming, or whether there are communication challenges, is unknown.

Respondents generally agreed that a broad range of impact indicators were needed and were important beyond bibliometrics and traditional academic outputs only (Fig. 1, Question 3). However, these were still regarded as the best short-term measures when biological or discovery science is a long-term venture. There was a sense that publication bibliometrics did not explain the whole story but at least were objective and quantitative tools. Most agreed or strongly agreed that broader downstream indicators of impact were important (Fig. 1, Question 4) but could be difficult to demonstrate and were not in the control of the researcher.

Selected free-text comments from cancer researchers are shown below.

“*Basic research will be where the major advances will come from for the diagnosis and treatment of cancer in the coming years. The ‘success’ of individual basic research projects when thinking only of impacting healthcare in the relatively short term is very hard to ascertain. It is the body of literature that basic research contributes to that provides the invaluable information that will be used to improve the outcomes for cancer patients. This takes longer than the period of a single grant, yet the funding of that single grant can have far reaching (global!) impact.*”

“*No system works. Comparison between disciplines are impossible.*”

“*While some types of research can be measured by the impact on patients, improved health and well-being, healthcare delivery and improving society, this does not apply to all types of research. An appropriate mix of academic and direct health outcomes are needed depending on the type of research that is undertaken.*”

“*The problem in Australia is that the most senior scientists are not participating in NHMRC* [National Health and Medical Research Council] *panels. They are acting as academy assigners when they should be reviewing on Panels. Researchers must show what it is that they have achieved. Relying on metrics such as number without a measure of quality like the impact factor, allows those that confuse activity with progress to prosper at the expense of those who push back the boundaries of ignorance. See recent PNAS paper from Harold Varmus* http://www.pnas.org/content/111/16/5773 *full on the current problems with our research system-note the discussion of the damage the current emphasis on translational research is causing to discovery. This is a direct product of consumer involvement and needs to be recognised.*”

“*The long lead time between discovering new ways to influence tumour progression, and their (possible) translation into new treatments, means that impact on patients is an impractical way to assess research impact. Discovery science is an incremental process and basic research can make a real contribution to future discoveries even though that contribution may be difficult to quantify. The difficulty in quantifying eventual impact should not be used as a reason to minimise support for discovery research.*”

“*Translational outcomes that are not reflected in publications should be highly valued.*”

“*Measurement of research impact needs to include a wide variety of assessments that include traditional publication and presentation assessments but also peer opinions, end user assessments and patterns of consistency across research areas that indicate long term development of sound strategies rather than scientific opportunism.*”

“*Observation and measurement usually influence the phenomenon that is being observed and measured. Researchers know this all too well. The people who survey researchers also need to know it. They have a responsibility to understand the incentives and disincentives they create. Good research is built on complex social systems of trust and co-operation as well as competition, and I am seeing many of the co-operative/trust systems undermined by the neurotic pursuit of quantifiable outcomes. ‘Not everything that counts can be counted, and not everything that can be counted counts.’ Above all, to count is to intervene. So intervene carefully.*”

“*Significance is a flexible issue depending upon who is assessing, very hard if too much weight is on consumer evaluation.*”

“*Quality better than quantity. Lots of cancer research in Australia, few do it at outstanding and internationally acclaimed levels and too few opportunities to translate research findings into meaningful clinical outcomes. Limited home grown biotech/pharmaceutical industry, limited venture capital. Limited options for start-up and spin offs.*”

Question 5 was open-ended and requested the researcher to nominate the most suitable outcome measure pertaining to their research field. Research publications and academic outputs dominated the responses and changes in clinical practice was mentioned in 10/14 responses by researchers involved in cancer treatment. Teaching and mentoring was viewed as important impact indicators while others, reduction in burden of disease, and patient days in hospital were infrequently nominated. Responses were elaborated on in many cases and two examples are:

“*Definitely academic* (*outputs*) *as noted above* (*including student completions*) *as it is basic research that provides a knowledge base that underpins healthcare* (*drug development, new diagnostic tests, etc.*) *but in and of itself will not ‘improve society’, ‘healthcare delivery’, etc. in the short term.*”

“*Research publications are crucial but they are not the full story. My research can and has had an impact through professional education and changes to policy (e.g. concerning the use of diagnostic tests). Research projects are also a means of training junior researchers, so these are also important (they are much more accessible these days), as are conference posters and presentations. A singular focus on peer reviewed publications can create perverse disincentives. Research has an important role to play in teaching and mentoring junior colleagues, and its influence is extended through these activities. ‘Not everything that counts can be counted’.*”

Having input into what is perceived to be the impact of their research was important to the respondents and engaging in this activity was acceptable. There were mixed responses to the question on the time spent collating information for demonstrating the impact of their research, with 40% (18/45) indicating that they spent too much time and 33% (15/45) indicating that they did not spend too much time on this task (Fig. 1, Question 7). This is likely to reflect the willingness of researchers to complete requests to provide impact indicators and shows the mix of expectations and work roles. Very senior level researchers may be time poor or engaged in teaching or clinical roles and the extra burden of this task perceived as too onerous. However, many appeared to be resigned to the idea that research impact activities were part of the job.

There were affirmative responses to their active role in grant reviewing and using judgement about the significance of a research funding proposal (42/45, 93%). The respondents may be well experienced in this area and view their qualifications as well-placed to perform this role, although one professor in the free text comments contradicted this by saying there were not enough scientists on review panels in Australia.

Mixed responses were received on the question of wthe perceived pressure to produce traditional academic outputs (i.e. publications, conference presentations, etc.; Fig. 1, Question 9). Some respondents confirmed they were under pressure while others emphasized the priority towards quality versus quantity of their outputs. However, in the follow-up question on autonomy of producing good science, many indicated they disagreed (27/45, 60%) that striving for research outcomes undermined their authority or autonomy in scientific research. This may show that the researchers were largely secure in their work environment and the research outputs they produced. Again, this may be influenced by the seniority of the respondents answering this survey.

Further comments from the open-ended answers, as indicated above, where all researchers completed this question, showed that the researchers:

- Believed that the purpose of impact activity should not be used for future funding decisions;
- Acknowledged the difficulties and pressures to produce good outputs but interference from stakeholders with the research process was unwelcome;
- Understood that the type of research will dictate the type of outcomes possible or those reasonable to quantify;
- Believed that quality of outputs or publications was a more important goal than quantity;
- Felt that there were few opportunities for advancing biological discoveries into clinical outcomes in Australia; and
- Felt that collecting research impact data was intervening in research because it created incentives and disincentives for researchers to engage in certain research activities and not others.

## Discussion

While there is increasing literature around impact evaluation, as well as the different models and challenges of such [1, 11–13], the results of this survey contribute to the under-investigated area of understanding researcher views about their role in impact evaluation. Recent work exploring researcher’s views of impact evaluation found that the term ‘impact’ has different meanings for researchers from different fields (humanities versus basic science) and researchers across fields have different motivations for conducting research (e.g. contribute to the social good, influence policy and practice, or advance career) [14]. The results of this survey extend this work by gaining a contemporary understanding of Australian cancer researcher’s views on their role in impact evaluation. It also has practical implications as these results may shape the way that funding agencies effectively engage with researchers in measuring impact. Understanding the views of cancer researchers in terms of the role they believe they should play in impact evaluation is an important element of a successful research impact evaluation framework.

CCNSW funds cancer researchers across a broad range of disciplines from basic science through to applied sciences and intervention projects. Due to the small sample size, sub-group analysis by type of research or career level did not produce meaningful outcomes. It was expected that we would find differences between career levels, with early career researchers more open to research impact evaluation and more accepting of this as their responsibility. Additionally, we were expecting to see differences between the types of research; in particular, it was expected that basic science researchers would have different views on impact evaluation and its relevance to their field compared to more applied researchers. This is a potential area for further investigation with a larger study population.

This survey has confirmed some of the conceptual challenges of research impact assessment, including attributing downstream outputs to a specific grant source when there is a time lag between funding and outputs and the choice of impact indicators when variation in the types of research undertaken occurs but where comparisons may be required. It is apparent from this study that a balance is required to source research impact indicators from researchers while also minimising the additional burden and perceived ‘interference’ with their research. As research impact assessment is now a fundamental activity for funding agencies and universities, there is a role for funding agencies to play in moving towards impact reporting and evaluation becoming standard practice while not increasing the burden for researchers too greatly. This debate around who ultimately should be responsible for tracking impact (researchers or funders) is a major challenge of research impact evaluation. Understanding researchers’ views on this issue helps to inform the development of a framework that will be acceptable to both researchers and funding agencies. While it is imperative to obtain researchers’ perspectives on and understanding of their impacts, it could be argued that there is an inherent conflict of interest perceived in a system in which those subject to evaluation are also the sole source of information. A research evaluation framework which distributes responsibility to both the researcher and funding agency may be the most ideal option. Incorporating existing processes like annual reporting, the onus of data collation could be, in the first instance, on funders rather than researchers. Principal researchers could have a more reduced role in impact assessment activities than previously planned (i.e. a supportive or validating role), and that the key assessment be performed externally by a dedicated evaluator with the skills and ability to complete the data synthesis and interpretation required. Additionally, as researchers are not always aware of their impact, there may be added value in feeding evaluation results back to researchers in this way.

It is not only funding agencies that require impact information but also research organisations such as universities. While some of the information may already be centralised or readily accessible (e.g. publication and patent databases), there are several systems, such as ResearchFish^®^, ORCID (Open Researcher and Contributor ID), and VV-Impact Tracker, which are available to assist researchers to report the outputs and impact of their research. With online systems such as ResearchFish® researchers are required to enter their data, and therefore the administrative reporting burden is on the researcher. The benefit of such a system would be if all or a majority of funding agencies sign up to an online system thereby reducing the burden on researchers to some extent, allowing researchers to report the same information across a number of funders, recording outputs once, and attributing these to multiple grants and/or funders. This would allow funders to gather information from researchers as the grant is ongoing, and for several years afterwards, building up a database of information that they can analyse to understand the progress, productivity and quality of their research portfolios. However, online systems such as ResearchFish® require significant financial investment from funding agencies over the long-term because research impact on policy and practice can often take decades in some cases. Importantly, the funding agency wishing to review this information still needs to invest resources in expertise to synthesise and interpret the reports provided from online tracking systems. It is also unclear how compliant researchers will be in reporting outputs and outcomes after the grant period has finished, when the report incentive (continual funding) has ceased.

A clear limitation of this survey was that it was small and the response rate was below 50%. Therefore, these findings should be viewed cautiously and may not be generalizable to the wider cancer research community. As completion of the survey was anonymous, the extent and influence of potential selection bias in the survey is unknown. Respondents may have been knowledgeable, interested or already engaged in this research impact work. Senior researchers are often on funding panels and therefore likely to have an informed understanding of why funders need to rely on researchers to provide this information. These researchers also provide an important link to stakeholders as they act as a conduit between the funders and the research community. To our knowledge, this is the first survey to assess the sentiments and practicalities of engaging in impact activities among researchers. Some questions may need improvements in their framing or tone and additional questions may be worthwhile, such as the researcher’s willingness to use an online system like ResearchFish^®^ or the researcher’s time spent on communicating the importance of research impact among junior team members.

Given that the results of this survey support previous research, in that the term ‘impact’ had different meanings for researchers across the cancer research continuum, it may be advisable that cancer funding bodies communicate their expectations early, at the grant acceptance stage, about the extent and types of data they expect grant recipients to collate. Expectations for basic science cancer researchers could be quite different from those of more applied cancer researchers, where funding agencies would require more traditional outputs for basic science researchers compared to broader impact expectations for clinical or public health researchers. It may also be advisable that funders engage with grant recipients to not only state their expectations but to communicate specifically why impact information is required and the value it has for the funder as a means of influencing researcher and donor behaviours and communications around such reporting policies.

## Conclusions

There is mixed support and acceptance among senior cancer researchers in Australia on their perceived role and engagement with research impact activities. Research impact evaluation needs to be a collaborative approach between researchers and funding organisations. Sole reliance on researchers for key information on research outputs is problematic. Requesting information from researchers could be minimalized and confined to the existing final reports and possible verification of completed evaluations.

## Abbreviation

CCNSW: Cancer Council New South Wales
